# TTANAD: Test-Time Augmentation for Network Anomaly Detection

**DOI:** 10.3390/e25050820

**Published:** 2023-05-19

**Authors:** Seffi Cohen, Niv Goldshlager, Bracha Shapira, Lior Rokach

**Affiliations:** Software and Information Systems Engineering, Ben-Gurion University, Beer Sheva P.O. Box 653, Israel; nivgold@post.bgu.ac.il (N.G.);

**Keywords:** NIDS, TTA, anomaly detection, time series

## Abstract

Machine learning-based Network Intrusion Detection Systems (NIDS) are designed to protect networks by identifying anomalous behaviors or improper uses. In recent years, advanced attacks, such as those mimicking legitimate traffic, have been developed to avoid alerting such systems. Previous works mainly focused on improving the anomaly detector itself, whereas in this paper, we introduce a novel method, Test-Time Augmentation for Network Anomaly Detection (TTANAD), which utilizes test-time augmentation to enhance anomaly detection from the data side. TTANAD leverages the temporal characteristics of traffic data and produces temporal test-time augmentations on the monitored traffic data. This method aims to create additional points of view when examining network traffic during inference, making it suitable for a variety of anomaly detector algorithms. Our experimental results demonstrate that TTANAD outperforms the baseline in all benchmark datasets and with all examined anomaly detection algorithms, according to the Area Under the Receiver Operating Characteristic (AUC) metric.

## 1. Introduction

Network anomaly detection plays a crucial role in defending against a wide range of cyber attacks, as modern cyber threats become increasingly sophisticated and persistent in evading detection systems. Intrusion detection (ID) is the core element for network security [1]. The main objective of ID is to identify abnormal behaviors and attempts caused by intruders in the network and computer system [2]. Network Intrusion Detection Systems (NIDS) combine information from sensors that monitor different network points around the organization’s network. The sensors monitor the incoming and outgoing traffic and can collect informative network features such as packet payloads, IP addresses, ports, number of bytes transmitted, and other network flow characteristics [3]. NIDS can be broadly categorized into two main groups: Signature-based NIDS and Anomaly-based NIDS. Signature-based NIDS are static in that the detection methods rely solely on a fixed set called a knowledge database, which needs to be updated over time and requires more human effort and time [4]. On the other hand, Anomaly-based NIDS are dynamic because after the normal state of the network is learned, they can detect any irregular and anomalous events [5]. The learning involves creating a baseline profile representing normal network behavior based on historical network traffic or a malicious-free network traffic snapshot. As a result, anomaly-based NIDS are considered the most popular detection method because they can detect unknown attacks (zero-day attacks) [6]. In real-world cyberspace tasks, storing, transferring, and processing the huge amount of data captured by the sensors is a big issue [7]. Sampling techniques have been proposed in several works [8,9,10] in order to cope with this challenge. These techniques aim at taking a portion of the data that gives the same characteristics as the whole dataset. Brauckhoff et al. [11] detailed the complete processing chain from packet capture to the generation of anomaly detection and included *temporal aggregation*, which extracts statistics such as mean, standard deviation, etc., from the data that arrives during a time window with a length of *T*. *Temporal aggregation* is applied to achieve further data compression and to transform the traffic trace into the observation timescale of interest for anomaly detection [11].

Test-time augmentation (TTA) is an application of data augmentation techniques on the test set. TTA techniques generate multiple augmented copies for each test instance, predicting each of them and combining the results with the original instance’s prediction [12]. Intuitively, TTA produces different points of view at inference time, thus predicting the given test instance more robustly. Data augmentation can improve the model’s performance without changing its architecture. However, it requires more training resources since more training data are used [13]. TTA, on the other hand, is more efficient than data augmentation in the training phase because retraining the model is not required. Several studies, mostly from the vision domain, have used various test-time augmentation techniques in their work [14,15].

The TTA is commonly used in image classification tasks to improve the performance of machine learning models by augmenting the test data. It has been shown to provide a significant boost in the predictive performance of various machine learning models. However, no previous works have utilized TTA for network anomaly detection, primarily because TTA has been predominantly applied to image and text data. The lack of application of TTA in network anomaly detection presents an opportunity to explore the potential benefits of this technique for enhancing the performance of NIDS.

In this paper, we propose a novel method, Test-Time Augmentation for Network Anomaly Detection (TTANAD), which utilizes test-time augmentation to improve network anomaly detection. By taking advantage of the temporal characteristics of traffic data, TTANAD generates temporal test-time augmentations on the monitored traffic data to create additional points of view when examining network traffic during inference. The experimental results demonstrate that TTANAD performs better on all benchmark datasets and all examined anomaly detection algorithms.

The main contributions of this work are as follows:

- We introduce TTANAD, a novel method that leverages test-time augmentation to improve the performance of network anomaly detection tasks, across various anomaly detection algorithms.
- Our work introduces the unique approach of generating synthetic augmentations based on temporal aggregation features at test time, without modifying or retraining the underlying models.

The remainder of this paper is organized as follows. Related work is given in Section 2. The proposed method is explained in detail in Section 3 and Section 3.3 describes our benchmark datasets, experiments, and experimental set. Results are detailed in Section 4. Finally, Section 5 concludes this paper and provides the prospect of future work.

## 2. Related Work

### 2.1. Network Anomaly Detection

Anomaly detection can be defined as identifying patterns in the data that do not conform to expected behavior in some context [16]. Anomaly detection modeling can be broadly categorized into several types of techniques: statistical methods, neighbor-based methods, and dimensionality-based methods [16]. In statistical methods, the low probability samples under the learned distribution will be considered as an anomaly. Neighbor-based methods assume that normal data has significantly more neighbors than anomalous data. Dimensionality reduction-based methods try to find an approximation of the data using a combination of attributes that capture the bulk of the variability in the data. Additionally, anomaly detection can be accomplished using reconstruction methods that reconstruct the input from latent space. The reconstruction error of anomalous instances will be higher as the model has been adapted to reconstruct only normal data [17]. Despite the progress in this field, detecting sophisticated attacks remains a significant challenge due to the evolving nature of threats and the increasing volume of network traffic. In our experiments, we used an Autoencoder as a reconstruction-based anomaly detector, an Isolation Forest as a statistical-based anomaly detector, and a Local Outlier Factor as a neighbor-based anomaly detector.

#### 2.1.1. Autoencoder-Based Anomaly Detection

A method was proposed by Dau [18] that uses a replicator neural network, also referred to as an autoencoder, for anomaly detection. It can work in both single and multiple-class settings. The network is trained to reconstruct only “normal” observations, so it is assumed that normal samples should have low reconstruction error. Conversely, anomalous samples are expected to have higher reconstruction error because the network is not trained to replicate them. Autoencoders have been extensively studied for network intrusion detection (NID) [19,20,21,22,23,24]. However, a major weakness of autoencoder-based anomaly detectors is their struggle to identify anomalies in complex or noisy data accurately. This is because autoencoders aim to reproduce the input data closely. However, if the input data are complicated or noisy, the autoencoder may fail to capture the underlying patterns, failing to identify anomalies. Our proposed method, TTANAD, is designed to enhance the performance of various anomaly detection algorithms, including autoencoders, by providing additional perspectives on the test data through temporal augmentations. By offering autoencoders more opportunities to detect anomalies, we aim to overcome their potential weaknesses in identifying sophisticated attacks. In our evaluation, we used an autoencoder-based anomaly detector to test our approach and examine the effectiveness of TTANAD in improving detection performance.

#### 2.1.2. Local Outlier Factor Anomaly Detection

The Local Outlier Factor (LOF) was proposed by Breunig [25] as an unsupervised anomaly detection technique that calculates the anomaly score based on the deviation of a data point’s local density compared to its neighbors. It classifies samples with significantly lower density than their neighbors as outliers. The method involves determining the local density of a sample using its k-nearest neighbors, and the LOF score of observation is calculated as the ratio of its k-nearest neighbors’ average local density to its own local density. Normal samples are expected to have a similar local density to their neighbors, while abnormal data are expected to have a much lower local density. LOF has been widely studied for network intrusion detection [25,26,27,28,29,30], but its internal density-based mechanism can make it less effective at detecting anomalies that are not well-separated from normal data points or are located in low-density regions of the data. Our proposed method addresses this weakness by providing augmented instances for each sample with different values. One of these augmented instances has a better chance of separating anomalies due to its feature. In addition to autoencoders, we also evaluate the effectiveness of our proposed method, TTANAD, by employing the LOF algorithm as one of the anomaly detectors in our experiments. This allows us to assess the performance improvements offered by TTANAD across different anomaly detection techniques.

#### 2.1.3. Isolation Forest Anomaly Detection

The Isolation Forest method, introduced by Liu [31], is a technique for identifying anomalies by constructing decision trees. The method works by randomly selecting a feature and splitting the values of the selected feature, resulting in partitions. Anomalies are instances with short average path lengths on the trees as they are less common and require fewer splits to separate them from regular observations. Despite being widely used for Network Intrusion Detection [32,33,34,35,36], Isolation Forests are prone to be impacted by outliers and instances that significantly differ from the rest of the data, leading to possible false positive or false negative results. The use of TTA should improve robustness by providing more points of view for each instance. The isolation forest algorithm is another anomaly detector that we incorporate as part of our experiments, similar to autoencoders and LOF.

### 2.2. Test-Time Augmentation

Test-time augmentation is the process of producing several enhanced copies of each sample in the test set, applying a prediction for each, then returning an ensemble of those predictions. TTA was extensively shown to improve results in many domains, most notably the vision domain. In Alexnet [15] the authors also applied TTA by averaging the predictions on ten randomly cropped parts of the inference image. Cohen et al. [37] proposed Test-Time Augmentation for the tabular anomaly Detection technique, a TTA-based method to improve anomaly detection performance on all kinds of tabular data. Shanmugam et al. [12] determine the augmentations used in TTA by setting an appropriate weight for each augmentation created. Their method significantly outperforms existing approaches by focusing on the factors influencing TTA augmentation and finding the optimal weight per augmentation. A study by Cohen et al. [38] presented state-of-the-art results using TTA to predict Intensive Care Unit (ICU) survival. Although TTA has been successfully applied to images, text, and tabular data, its application to network anomaly detection has not been extensively explored. This research aims to fill this gap by proposing a TTA technique specifically designed for network anomaly detection tasks.

## 3. Materials and Methods

In this section, we first provide a problem formulation for network traffic anomaly detection and the temporal aggregation technique, which we later utilize to describe our proposed approach—TTANAD. Our method proposes a novel approach for creating meaningful augmentations in such a way that is specifically appropriate in the domain of time-series anomaly detection. As such, TTANAD is used to improve the inference performance from the data perspective instead of the modeling, i.e., the architectural perspective. Later, we describe the experiments performed and the setup used to evaluate TTANAD. Note that TTANAD is agnostic to the anomaly detector that is used in the pipeline. As a result, our extensive experimental study presents how we adapted several anomaly detectors to show generalization. We utilize the TTANAD on Network anomaly detection tasks to evaluate our approach.

### 3.1. Temporal Aggregation Formulation

In our work, we aggregate the raw time-series network traffic data with a sliding window. This approach is described in previous works [11,39] as a preprocessing step called *temporal aggregation* (we use this term), and as a pre-filtering phase. Temporal aggregation is used to cope with the high volume and velocity of the captured data from the network sensors. This approach extracts high-level information from the raw data features, such as basic statistical measures, utilizing aggregations with a fixed-size sliding window technique that takes advantage of the natural temporal characteristics of data. These extracted statistics, obtained by the aggregations, are then passed to an anomaly detector.

Formally, a sequence of raw network traffic data with *n* samples is defined as,

$$\begin{array}{c} X={\left[\begin{array}{cccccc} x_{1} & x_{2} & \cdots & x_{i} & \cdots & x_{n} \end{array}\right]}^{T} \end{array}$$

$x_{i}\in R^{d}$, where $x_{i}$ is a vector of *d* real-valued features that represent each sample in the dataset. Here, *d* signifies the number of features captured for each sample, and R denotes the set of real numbers. The temporal aggregation is defined with a window size *w* and step size *s*, such that when convolving, each window consists of $X_{t:t+w}={\left[\begin{array}{cccc} x_{t} & x_{t+1} & \cdots & x_{t+w} \end{array}\right]}^{T}$, where *t* is an arbitrary timestamp such that the previous window is $X_{t-s:t-s+w}$. Note that $X\in R^{n\times d}$ and $X_{t:t+w}\in R^{w\times d}$. $X_{t:t+w}^{k}$ is defined as we refer only to feature *k* from all the samples in that sequence.

A set of *m* aggregators $G=\{G_{1},G_{2},\cdots ,G_{i},\cdots ,G_{m}\}$ operate on each window $X_{t:t+w}$:

(1) $$\begin{array}{c} \Phi_{t:t+w}^{G}=G\left(X_{t:t+w}\right)=\overset{m}{\underset{i=1}{⋃}}G_{i}\left(X_{t:t+w}\right) \end{array}$$

$\Phi_{t:t+w}^{G}$ is an aggregated window starting at timestamp *t* with window size *w* over the G aggregators set. The operation of a single aggregator is defined as follows:

(2) $$\begin{array}{c} G\left(X_{t:t+w}\right)=\left[\begin{array}{cccc} G(X_{t:t+w}^{1}) & G(X_{t:t+w}^{2}) & \cdots & G(X_{t:t+w}^{d}) \end{array}\right] \end{array}$$

Note that $\Phi_{t:t+w}^{G}\in R^{1\times m\cdot d}$ because the aggregation operates over the time dimension and the union operates over the feature dimension (Only one direction of inner windows is created for the first and last aggregation windows).

In our work, we used three aggregators: minimum, maximum, and standard deviation denoted as $G_{min}$, $G_{max}$, and $G_{std}$, respectively; using Equation

$$\begin{array}{cc} \; & G_{min}\left(X_{t:t+w}\right)=\left[\begin{array}{ccc} min\{X_{t:t+w}^{1}\} & \cdots & min\{X_{t:t+w}^{d}\} \end{array}\right]\\ \; & G_{max}\left(X_{t:t+w}\right)=\left[\begin{array}{ccc} max\{X_{t:t+w}^{1}\} & \cdots & max\{X_{t:t+w}^{d}\} \end{array}\right]\\ \; & G_{std}\left(X_{t:t+w}\right)=\left[\begin{array}{ccc} std\{X_{t:t+w}^{1}\} & \cdots & std\{X_{t:t+w}^{d}\} \end{array}\right] \end{array}$$

where $std\{X_{t:t+w}^{k}\}$ denotes calculating the standard deviation over the sequence $X_{t:t+w}^{k}$:

$$\begin{array}{c} std\left\{X_{t:t+w}^{k}\right\}=\sqrt{\frac{\sum_{i=t}^{t+w}{\left(X_{i}^{k}-\mu \right)}^{2}}{w}},\;\mu =\frac{\sum_{i=t}^{t+w}X_{i}^{k}}{w} \end{array}$$

So, in our case, where $G=\{G_{min},G_{max},G_{std}\}$, the generic definition of $\Phi_{t:t+w}^{G}$ from Equation (1), is now defined as follows:

$$\begin{array}{cc} \; & \Phi_{t:t+w}^{G}=G_{min}\left(X_{t:t+w}\right)\cup G_{max}\left(X_{t:t+w}\right)\cup G_{std}\left(X_{t:t+w}\right) \end{array}$$

(3) $$\begin{array}{cc} \Phi_{t:t+w}^{G} & =\left[\begin{array}{ccc} min\{X_{t:t+w}^{1}\} & max\{X_{t:t+w}^{1}\} & std\{X_{t:t+w}^{1}\} \end{array}\right.\\ \; & \left.\begin{array}{cccc} \cdots & min\{X_{t:t+w}^{d}\; & max\{X_{t:t+w}^{d}\; & std\{X_{t:t+w}^{d}\; \end{array}\right] \end{array}$$

thus now $\Phi_{t:t+w}^{G}\in R^{1\times 3d}$.

### 3.2. Temporal Aggregation-Based TTA

Given a time-series network traffic *X* and a set of aggregators G=Gmin,Gmax,Gstd, we define Gmin as the minimum value in *X*, Gmax as the maximum value in *X*, and Gstd as the standard deviation value of *X*. We extend the temporal aggregation to the test phase as a test-time augmentation technique to produce a more diverse and robust final prediction [12]. TTANAD produces augmentations by creating synthetic “inner” aggregation windows with a window size $w^{\prime }=w$ and a step size $s^{\prime }=1$ (i.e., step size of 1 and same window size used to create the original aggregation windows). Formally, the augmented inner windows for an arbitrary test window $X_{t:t+w}$ with $s^{\prime }=1$ and $w^{\prime }=w$ are as follows:

(4) $$\begin{array}{c} TTA\left(X_{t:t+w}\right)=\{X_{t-w+1:t+1},X_{t-w+2:t+2},\cdots ,\\ X_{t-1:t-1+w},X_{t+1:t+1+w},X_{t+2:t+2+w},\\ \cdots ,X_{t+w-1:t+2w-1}\} \end{array}$$

Now, we can define TTA with aggregations:

(5) $$\begin{array}{c} \Phi_{TTA(X_{t:t+w})}^{G}=\left[\begin{array}{ccc} — & \Phi_{t-w+1:t+1}^{G} & —\\ — & \Phi_{t-w+2:t+2}^{G} & —\\ \; & \vdots & \\ — & \Phi_{t-1:t-1+w}^{G} & —\\ — & \Phi_{t+1:t+1+w}^{G} & —\\ — & \Phi_{t+2:t+2+w}^{G} & —\\ \; & \vdots & \\ — & \Phi_{t+w-1:t+2w-1}^{G} & — \end{array}\right] \end{array}$$

Finally, a simple average operation is used, considering all the TTA’s predictions, ${\hat{y}}_{\Phi_{TTA(X_{t:t+w})}^{G}}$ as well as $\Phi_{t:t+w}^{G}$ (i.e., the original test aggregated window) prediction, ${\hat{y}}_{\Phi_{t:t+w}^{G}}$, to obtain the final prediction:

(6) $${\hat{y}}_{final}=\frac{\left\{{\hat{y}}_{\Phi_{t:t+w}^{G}}\right\}\cup {\hat{y}}_{\Phi_{TTA(X_{t:t+w})}^{G}}}{∥TTA∥+1}$$

**Figure 1:**
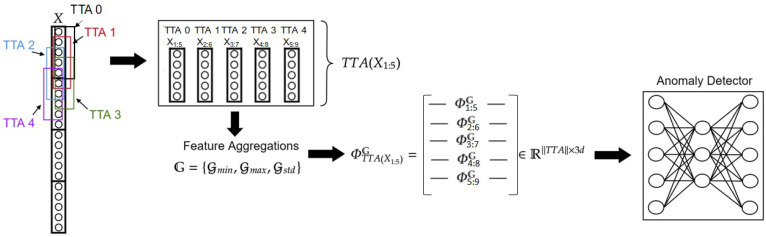
Temporal aggregation-based TTA: Creating more samples using inner windows with a stride of 1, then extracting temporal features using the defined aggregators. We produce a prediction for the original window and the augmentations using the anomaly detector, then calculate the final prediction using the average of all predictions.

The motivation behind setting $w^{\prime }$ as the same as *w*, is that the synthetic aggregation windows created will be constructed from the same amount of samples as the original windows. We decided to set $s^{\prime }=1$ in order to create the maximum number of inner windows possible because more information could be exploited by the model. Figure 1 illustrates an example with w=5, s=5. As demonstrated, setting $w^{\prime }=w=5$, $s^{\prime }=1$ results in the creation of 4 TTAs for the first original aggregation window. Note that, for the first and the last test aggregation windows ∥TTA∥=w−1 while for the other test aggregation windows ∥TTA∥=2(·w−1).

The entire schema of using TTANAD can be summarized as follows at a higher level. First, the raw time-series network traffic data are aggregated using *temporal aggregation*. We then train an anomaly detector using the aggregated data and predict anomalies utilizing TTANAD as a test-time augmentation method. Intuitively, TTANAD could create augmentations that would give an ensemble of new perspectives to the anomaly detector at inference, which would result in better performance.

### 3.3. Experiments

In this section, we describe the experiments conducted to evaluate the impact of TTANAD on anomaly detection. All of the evaluated algorithms, anomaly detectors, datasets, and experimental setups are explained in the subsections below.

#### 3.3.1. Data

Three datasets of raw network packets were used for evaluation: Two datasets from the Canadian Institute for Cybersecurity (https://www.unb.ca/cic/datasets/, accessed on 16 May 2023), and the UNSW-NB15 dataset from the Cyber Range Lab of UNSW Canberra (https://research.unsw.edu.au/projects/unsw-nb15-dataset/, accessed on 16 May 2023):

- **CIC-IDS2017**—The CIC-IDS2017 [40] dataset includes eight different files containing five days’ normal and malicious traffic data. Combining these files results in roughly 3 million instances and 83 features with 15 labels—1 normal and 14 attack labels.
- **CSE-CIC-IDS2018**—The CSE-CIC-IDS2018 [40] dataset contains about 16 million instances collected over ten days, with roughly 17% of the instances compromised of malicious traffic.
- **UNSW-NB15**—The UNSW-NB15 [41] dataset was created by the IXIA PerfectStorm tool for generating a hybrid of real modern normal activities and synthetic contemporary attack behaviors. This dataset contains about 2.5 million instances and 49 features.

#### 3.3.2. Preprocessing

In our experiments, we first preprocessed the datasets to ensure that they were suitable for the anomaly detection algorithms. This preprocessing involved the following steps:

- **Integrate and Sort:** We first loaded the data for the evaluated datasets, concatenating all of the provided files for each dataset and ordering the instances by their timestamp in ascending order.
- **Data Cleaning:** We removed any duplicate records and checked for inconsistencies in the dataset. If any inconsistencies were found, such as missing values, we either imputed them using appropriate statistical techniques or removed the corresponding records.
- **Temporal Feature Extraction:** Using a window with a size *s*, we are sliding it with a stride equal to *s* and aggregating the features of the instances in each window. The aggregation contains extracting minimum, maximum, and standard deviation for each feature.
- **Feature Scaling:** To ensure that all features were on the same scale, we standardized the numerical features using z-score normalization.
- **Data Splitting:** We time-based split to train and test sets using a 70–30% ratio, respectively.

It is important to note that our preprocessing steps were designed to prepare the datasets for the anomaly detection algorithms without introducing any biases or data leakage. If no inconsistencies were found during the data cleaning process, we continued with the remaining preprocessing steps to ensure the data were in a suitable format for our experiments.

**Figure 2:**
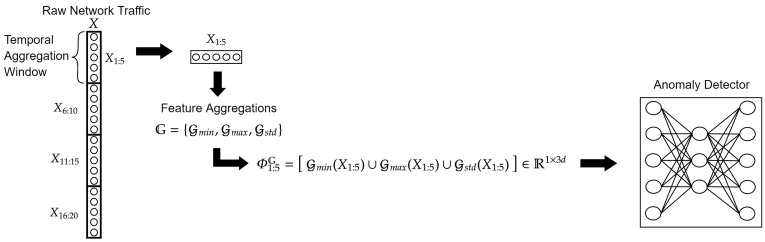
Temporal Aggregation: extracting temporal features using the minimum, maximum, and standard deviation aggregators with a window size and step size of 5. The extracted features are forwarded to the anomaly detector.

The anomaly detection schema with the described temporal aggregation formulation can be seen in Figure 2.

#### 3.3.3. Compared Algorithms

For each dataset, we inferred the anomaly detector using two methods: (1) using the standard test phase (w/o TTA) as the baseline for our method, and (2) a test phase with our suggested technique (TTANAD), that is, utilizing test-time augmentations generated by TTANAD for each test instance. Due to the lack of previous work utilizing TTA in the domain of network anomaly detection, a vanilla test phase is our only baseline.

#### 3.3.4. Evaluated Anomaly Detectors

We used three different anomaly detectors for each evaluated dataset. We compared the anomaly detector algorithm and the temporal aggregation window size, in order to demonstrate the superiority of TTANAD over its baseline. Different types of anomaly detectors were chosen, such as reconstruction-based, density-based, and tree-based algorithms.

#### 3.3.5. Experimental Setup

Sakurada et al. [42] proposed using autoencoders as a dimensional reduction-based approach to detect subtle anomalies. In our experiments, we utilize an autoencoder as an anomaly detector. We trained the autoencoder in the same way for all datasets. After tuning, the architecture of the autoencoder is as follows: input layer with a number of neurons corresponding to the number of features, a hidden layer with 64 neurons, latent space with 16 neurons, a hidden layer with 64 neurons, and an output layer with the same size of the input layer. All the hidden layers are followed by ReLU activation. The autoencoder was trained for 300 epochs and with a batch size of 32. We used Adam optimizer with default values of parameters (i.e., $\beta_{1},\beta_{2}$). The loss function that was used is Mean Squared Error (MSE).

The isolation forest anomaly detector was trained using 150 estimators (in the ensemble) while overriding the default value of max_samples with 0.94, meaning we take 94 % of the training data (without bootstrapping) to train each estimator. Moreover, each estimator was trained using the whole feature space.

The Local Outlier Factor detects anomalies based on the density of a sample within its neighborhood. As a sample with a density measure substantially lower than its neighbors, this sample is considered an anomaly. As a result, the main two hyperparameters in this anomaly detector are the neighborhood size n_neighbors, and the distance metric metric. We used the standard Euclidean distance metric in our experiments while optimizing the n_neighbors across the range [2,150].

We reported the results on the presented datasets when varying the aggregation window size with values {3,4,and 5}. Note that when changing different aggregation window sizes, both the baseline and TTANAD results change because the window size affects both the aggregation operation result (varying window sizes) and the amount of test-time augmentations.

After calculating the anomaly score of every sample in the test set, we measured the Area Under the Curve (AUC) for the generated ROC curve. We chose to measure the AUC because it is agnostic to the threshold (anomaly score) and a suitable metric in anomaly detection [43,44].

The experiments were implemented using TensorFlow (https://www.tensorflow.org/, accessed on 16 May 2023) 2.x and RAPIDS (https://rapids.ai/, accessed on 16 May 2023) with CUDA 11.4 using Nvidia GeForce RTX 2080 Ti with 11 G memory and 32 G RAM on a CentOS machine. The benchmark datasets, the anomaly detector, and TTANAD’s reproducible source are publicly available (https://github.com/nivgold/TTANAD, accessed on 16 May 2023).

## 4. Results

The results of the methods are presented in Table 1. These results demonstrate that all network anomaly detectors perform better in all experiments when using TTANAD. It is important to note that even a small AUC margin of $10^{-3}$ can be significant when detecting “non-trivial” anomalies, particularly in the domain of network traffic, where dealing with high volume data leads to a considerable number of detections. TTANAD outperforms the baseline (i.e., without TTA) on all compared datasets and for all window sizes. The LOF anomaly detector experienced the most significant improvement, likely due to its initially poor baseline results. Conversely, the autoencoder and isolation forest anomaly detectors had the lowest average TTANAD improvements, despite achieving the best performance among the other two anomaly detectors.

We can conclude that sliding window-based augmentations enable the final prediction to obtain a wider perspective on the test instance, resulting in more robust and reliable predictions. This, in turn, helps the model achieve higher performance in detecting network traffic anomalies. Overall, the TTANAD method improved AUC results by 2.47% on the CIC-IDS2017 dataset, 0.6% on the CSR-CIC-IDS2018 dataset, and 1.6% on the UNSW-NB15 dataset. The CIC-IDS2017 and CSE-CIC-IDS2018 datasets offer a suitable representation of network traffic domains [45]. For a more comprehensive experimental validation, we also included the UNSW-NB15 dataset.

### Discussion

We have presented the AUC scores of our proposed TTANAD method and the baseline methods (i.e., without TTA) on three benchmark datasets (CIC-IDS2017, CSE-CIC-IDS2018, and UNSW-NB15) using three different anomaly detection algorithms (autoencoder, isolation forest, and local outlier factor). A deeper analysis of the results is necessary to understand the performance improvements achieved by TTANAD across various scenarios. For the CIC-IDS2017 dataset, TTANAD outperformed the baseline methods in all three anomaly detection algorithms, with the most significant improvement observed in the local outlier factor algorithm. This improvement can be attributed to the relatively poor baseline performance of the local outlier factor algorithm, leaving more room for enhancement. In the case of the CSE-CIC-IDS2018 dataset, improvements were relatively modest, with the highest improvement observed for the isolation forest algorithm. The smaller improvements could be due to the dataset’s nature or the already high performance of the baseline methods.

For the UNSW-NB15 dataset, TTANAD demonstrated significant improvements across all three anomaly detection algorithms. The autoencoder and isolation forest algorithms, which already had high baseline performance, showed substantial improvements, suggesting that TTANAD is particularly effective in this dataset.

These results indicate that the proposed TTANAD method can enhance the performance of various network anomaly detection algorithms across different datasets. The sliding window-based augmentations provide a broader perspective on the test instances, leading to more robust and reliable predictions, and ultimately improving the detection of network traffic anomalies.

## 5. Conclusions

Network anomaly detection plays a critical role in identifying abnormal patterns within network traffic. In this paper, we presented TTANAD, a novel test-time augmentation (TTA) technique aimed at enhancing the performance of various network traffic anomaly detection algorithms. The key advantage of our approach is that it does not require modifying or retraining the underlying anomaly detector models. Instead, TTANAD generates synthetic augmentations by leveraging temporal aggregation features during the test phase. To the best of our knowledge, no previous studies have applied test-time augmentation to network anomaly detection or tabular time series data. Our comprehensive experiments demonstrated that TTANAD consistently improved the predictive performance across multiple benchmark datasets and a diverse set of anomaly detection algorithms. This highlights the potential of our proposed method in advancing state-of-the-art network anomaly detection. As a direction for future work, we propose extending our methodology to supervised Signature-based Network Intrusion Detection Systems (NIDS). Additionally, incorporating a wider range of anomaly detection algorithms and comparing the results directly with existing literature will further validate the effectiveness of TTANAD and help identify areas for improvement or specific use cases where it performs particularly well.

## Figures and Tables

**Table 1:** AUC results on the evaluated datasets.

Method		CIC-IDS2017			CSE-CIC-IDS2018			UNSW-NB15		
Temporal Aggregation Window		T = 3	T = 4	T = 5	T = 3	T = 4	T = 5	T = 3	T = 4	T =5
Autoencoder	w/o TTA	0.754	0.769	0.767	0.925	0.921	0.922	0.989	0.987	0.985
	TTANAD	**0.760**	**0.782**	**0.773**	**0.930**	**0.926**	**0.929**	**0.995**	**0.994**	**0.992**
Isolation Forest	w/o TTA	0.813	**0.813**	0.870	0.948	0.948	0.944	0.889	0.921	0.909
	TTANAD	**0.819**	**0.813**	**0.884**	**0.950**	**0.950**	**0.947**	**0.922**	**0.946**	**0.930**
Local Outlier Factor	w/o TTA	0.654	0.675	0.701	0.665	0.651	0.708	0.640	0.937	0.836
	TTANAD	**0.6984**	**0.764**	**0.747**	**0.684**	**0.666**	**0.710**	**0.649**	**0.941**	**0.887**

## Data Availability

Three datasets of raw network packets were used for evaluation. The CIC-IDS2017 and CSE-CIC-IDS2018 datasets from the Canadian Institute for Cybersecurity are available at https://www.unb.ca/cic/datasets, accessed on 16 May 2023, and the UNSW-NB15 dataset from the Cyber Range Lab of UNSW Canberra is available at https://research.unsw.edu.au/projects/unsw-nb15-dataset, accessed on 16 May 2023.

## Abbreviations

The following abbreviations are used in this manuscript:

TTANAD	Test-Time Augmentation for Network Anomaly Detection
NIDS	Network Intrusion Detection Systems
TTA	Test Time Augmentation
ID	Intrusion Detection
